# Evaluation of High Vacuum Flavor Extraction Device as a Novel Technique for the Extraction of Volatile Compounds

**DOI:** 10.3390/foods13193206

**Published:** 2024-10-09

**Authors:** Mingyuan Liu, Jie Zhou, Jingkai Qin, Zhongyi Qin, Jiequn Jiang, Futian Yu, Mei Chen, Xiaoling Liu, Meishuo Zhang

**Affiliations:** 1 Department of Food Science, Guangxi University, No. 100, Daxue Road, Nanning 530004, China; jacoblmy@outlook.com (M.L.); zj111208@163.com (J.Z.); 17396752363@163.com (J.Q.); 1837715449@139.com (Z.Q.); 18815816692@163.com (J.J.); yufutian2012@163.com (F.Y.); 1668526568@163.com (M.C.); meishuozhang@163.com (M.Z.); 2 Key Laboratory of Deep Processing and Safety Control for Specialty Agricultural Products in Guangxi Universities, Education Department of Guangxi Zhuang Autonomous Region, Nanning 530004, China

**Keywords:** high vacuum extraction (HVE), gas chromatography–flame ionization detection (GC-FID), volatile compounds, sensory analysis, fried tilapia mince, extraction techniques, flavor compound analysis

## Abstract

In this study, a high vacuum flavor extraction (HVE) device was developed to address the limitations of traditional extraction methods, such as extended extraction times and artifact generation during high-temperature processes. Firstly, the repeatability and precision of the HVE method were evaluated through quantitative analysis of twelve volatile odor compounds across seven replicate extractions using gas chromatography–flame ionization detection (GC-FID). The results showed that the HVE system achieved a mean relative standard deviation (RSD) of 11.60 ± 1.79% and a recovery rate of 90.55 ± 4.56%, demonstrating its precision and reproducibility. Secondly, the performance of HVE was compared with solvent-assisted flavor evaporation (SAFE) and simultaneous distillation–extraction (SDE) for extracting flavor compounds from fried tilapia mince. The results indicated that HVE was more effective, particularly in extracting aldehydes and pyrazines, which are key contributors to the flavor profile. Finally, sensory evaluations supported these findings, showing that the odor profiles obtained through HVE were most similar to the original sample, with a similarity score of 72.55%, compared to 69.25% for SAFE and 60.29% for SDE. These findings suggest that HVE is a suitable method for the extraction and analysis of volatile compounds in complex food matrices such as fried tilapia mince.

## 1. Introduction

Fried foods, such as fried tilapia mince, derive their distinctive flavors primarily from a variety of volatile compounds, including low molecular weight aldehydes, ketones, acids, and esters. The types and concentrations of these volatile compounds dictate the aromatic characteristics of food, such as fragrant, roasted, and fresh aromas. These flavor characteristics directly influence market acceptance and consumer preferences for food products [1]. In the field of food science, the study of volatile compounds extends beyond flavor analysis; it also encompasses the storage stability, quality changes, and development of new products. For instance, research into specific volatile compounds produced during the frying process has enabled food engineers to optimize frying conditions, reduce the formation of harmful substances, enhance nutritional value, and improve the safety of food products. Concurrently, the generation of flavor substances during food processing has been examined [2]. For example, the preparation of fish mince gel was accompanied by the production of a significant amount of characteristic aroma compounds. Therefore, flavor substances generated during food processing could serve as an indicator to regulate the techniques employed in processing, thereby enhancing the quality of food [2]. Furthermore, structures within food, such as proteins, are able to adsorb these flavor compounds, thereby enabling the characteristic aromas to persist and allowing the food to maintain its distinctive fragrance over an extended period [3]. As consumer attention increasingly focuses on health and natural foods, and as demands for food quality escalate, the study of natural spices and additives in foods has become ever more crucial. By analyzing and simulating the volatile compounds in natural foods, healthier and more sustainable food additives could be developed to meet market demands [4]. Consequently, accurate and comprehensive analysis of volatile compounds is of paramount importance for understanding food flavors, enhancing food quality, guiding food processing, and facilitating the development of new products.

In the analysis of volatile compounds in fried foods, traditional solvent extraction and concentration methods face numerous challenges. Firstly, these methods typically require high extraction temperatures, which could lead to the loss or degradation of thermosensitive compounds. For instance, compounds containing volatile aromas are highly susceptible to decomposition at elevated temperatures, thereby affecting the final analysis and evaluation of flavors. Secondly, the extraction and concentration processes are time-consuming, which implies lower efficiency and higher costs in industrial applications. In the rapidly evolving food industry, enhancing analytical speed and reducing costs are pressing issues that need to be addressed [5]. Moreover, as most target compounds are low-boiling-point substances, the efficiency of separating high-boiling-point compounds is low, which can affect the accuracy and comprehensiveness of the final detection results. Conventional extraction methods, such as simultaneous distillation–extraction (SDE), have served as widely used techniques for the extraction of flavor substances [6]. SDE requires operation in a prolonged high-temperature environment, inevitably leading to the formation of additional substances, which could affect the odor profile of the extracted flavor substances [7]. Solid phase microextraction (SPME), as a novel method for the extraction of flavor substances, relies on the selective adsorption of the adsorbent materials. Due to the inability to conduct direct and effective sensory experiments, it is challenging to verify the odor profiles of the odor compounds extracted, thereby posing obstacles to the study of food flavors. Solvent-assisted flavor evaporation (SAFE) is a novel method that depends on a high vacuum environment to extract volatile flavor substances [8]. However, its expensive cost and complex design make it difficult to clean, thus causing further complications in scenarios requiring continuous extraction [9]. Therefore, finding a method capable of effectively extracting these compounds with diverse characteristics is crucial for enhancing the comprehensiveness and accuracy of food analysis.

To address these issues, this article describes the innovative design and development of a high vacuum flavor extraction (HVE) device that operates under a high vacuum environment, significantly lowering the boiling points of volatile compounds. This allows for effective extraction at lower temperatures, thus reducing the loss of thermosensitive substances. The device underwent precision and reproducibility tests using gas chromatography–flame ionization detection (GC-FID) to ensure it met the experimental requirements. To evaluate the effectiveness of the high vacuum flavor extraction device in extracting volatile compounds from fried tilapia mince, extractions were performed using three different methods: SAFE, HVE, and SDE. Subsequently, fourteen compounds in the extracts were quantitatively analyzed using GC-FID. Sensory evaluation was employed to assess the odor profiles of fried tilapia mince extracted by these methods to determine the most suitable method for extracting aroma substances. The experimental results indicated that the HVE method performed excellently and may become a new method for the study of food aromas.

## 2. Materials and Methods

### 2.1. Materials and Chemical Reagents

For this study, tilapia (*Oreochromis mossambicus*) was procured from the Luban Road Market in Nanning. After the removal of the skin and viscera, the muscle tissue was minced using a 5 mm sieve and then sealed in plastic bags and stored at −20 °C (to be utilized within one week). The soybean oil used for frying was supplied by China Food Group Co., Ltd. (Beijing, China). Analytical standards (>97%) were acquired from Sigma Company (St. Louis, MO, USA).

### 2.2. Preparation of Fried Tilapia Mince

The preparation of the tilapia mince gel was slightly modified from the method described by Yueqi An [2]. After slaughtering, the tilapia meat and skin were minced using a grinder and then frozen for 6 h at 4 °C. Subsequently, 2.5% sodium chloride was added and thoroughly mixed before being molded into 10 mm × 10 mm × 10 mm cubes. A total of 20 g of this mixture was placed into a 100 mL quartz test tube, to which 60 mL of soybean oil was added. Dimethyl silicone oil was preheated to 160 °C and maintained for 30 min. The test tubes containing the soybean oil and tilapia meat were then immersed in the dimethyl silicone oil and fried for 15 min, after which the quartz test tubes were rapidly cooled in crushed ice. The cooled fried tilapia mince was then filtered through three layers of cheesecloth to remove the fat, and the solid material was transferred to a 50 mL centrifuge tube. The material was centrifuged at 5000 rpm under room temperature conditions for 15 min to remove residual fat, and the resulting solid material was transferred to a 25 mL amber sample bottle for subsequent analysis [1].

### 2.3. Manufacturing and Features of HVE

**Figure 1:**
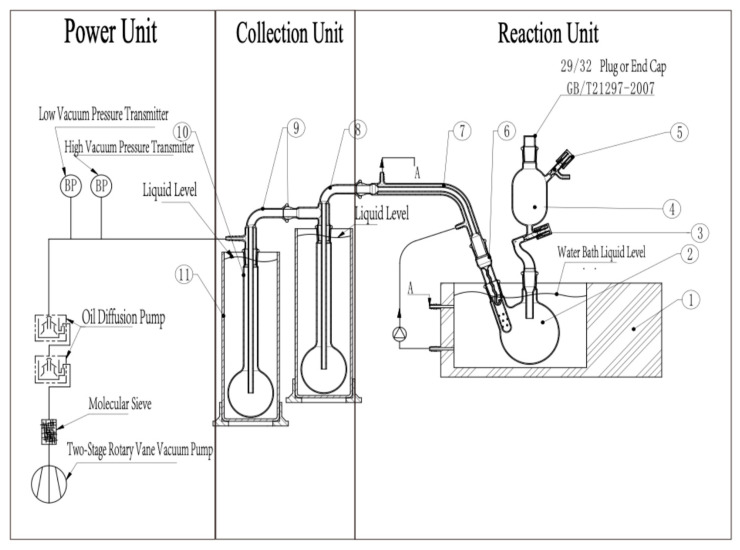
Schematic diagram of HVE (1—super thermostat system, 2—reaction chamber, 3—high vacuum valve, 4—high vacuum separating funnel, 5—high vacuum valve, 6—liquid stop tube, 7—constant temperature transfer tube, 8—collection tube, 9—collection tube, 10—collection chamber, 11—ultra-low temperature cold trap).

The HVE device, as illustrated in Figure 1, prominently featured three main units: the evaporation unit, the collection unit, and the power unit. Each played a crucial role in the overall functionality of the system.

The evaporation unit was engineered with an evaporation chamber, a liquid stop tube, a constant temperature transfer tube, and a super constant temperature system. The inlet of the evaporation chamber connected to the liquid inlet pipe of an adding funnel, branching off to incorporate a high vacuum valve. This design facilitated the introduction and vaporization of substances extracted with solvents under high vacuum conditions, ensuring that volatile compounds were efficiently evaporated. A specially designed liquid stop tube at the outlet of the evaporation chamber prevented boiling liquids from directly entering the constant temperature transfer tube, utilizing a serpentine-shaped glass tube with a 4/5 circular hook at its lower part to restrict passage to only gaseous substances.

The collection unit included a cold trap and a collection chamber, the latter of which was submerged in a cold trap filled with a low-temperature liquid to maintain a cold environment essential for condensing the volatile substances. The collection chamber was interconnected through collection pipes to the constant temperature transfer tube, which facilitated the transfer of collected volatiles from the evaporation unit.

The power unit consisted of an oil-sealed rotary vane primary pump and a secondary pump. The air intake of the primary pump was connected to the air outlet of the secondary pump, establishing a continuous flow system. The secondary pump, connected to the outlet of the cold trap, included both an oil diffusion pump and a molecular pump. This arrangement was critical for maintaining the high vacuum necessary for effective evaporation and collection. The power unit was capable of sustaining the system at a high vacuum state of 0.001 Pa. The cold trap featured dual systems using dry ice and liquid nitrogen, housed in a Dewar flask to ensure optimal insulation and efficiency.

When the high vacuum flavor extraction (HVE) device is operational, it initially utilizes the power unit to evacuate the internal atmosphere to a high vacuum state. Subsequently, the super constant temperature system is employed to maintain the temperature of the constant temperature reaction pool and the transfer pipelines within an optimal range. Following this, samples are introduced into the vacuum system via an adding funnel in conjunction with a high vacuum valve. Finally, after being transferred under high vacuum conditions, the target components are collected in the cold trap.

### 2.4. Precision and Reproducibility Experiment for HVE

According to ISO 5725-1:2023 and following the method described by Dexin Jiang [10,11], the precision and reproducibility of the HVE were determined. The preparation process for the mixed standard used in the precision and reproducibility experiment was as follows: 2,3,5-trimethylpyrazine (25 mg), 2,3-dimethyl-5-ethylpyrazine (25 mg), 2,3-dimethylpyrazine (25 mg), 2-n-hexylthiophene (30 mg), dimethyl trisulfide (25 mg), (E)-2-octenal (25 mg), (E,E)-2,4-decadienal (20 mg), benzaldehyde (15 mg), trans-2-nonenal (40 mg), hexanal (25 mg), hydroxyacetone (30 mg), and D-pantolactone (25 mg) were added to a 1 L volumetric flask, brought to volume with dichloromethane, and thoroughly mixed. The solution was then transferred into blue-capped bottles with polytetrafluoroethylene lids and stored at −20 °C. Following this method, two batches were prepared, and two graduate students from our laboratory extracted the compounds at different times using gas chromatography for detection and quantitative analysis.

### 2.5. Experiment on the Extraction and Separation of Volatile Compounds in Tilapia

#### 2.5.1. Preliminary Extraction of Key Volatile Flavor Compounds from Fried Tilapia Mince Using Accelerated Solvent Extraction Device (ASE)

An E-916 accelerated solvent extraction device was utilized for the extraction, with slight modifications to the method described in the literature [12]. A total of 40 g of anhydrous sodium sulfate was added to 40 g of fried tilapia meat, thoroughly mixed, and then placed into a pressure vessel. Nitrogen gas was used as a protective atmosphere. At a temperature of 50 °C and a pressure of 120 bar, 20 mL of dichloromethane was used for extraction for 10 min, followed by a 1 min equilibration. This process was repeated three times with each cycle using 10 mL of dichloromethane for a 2 min rinse. Subsequently, at a temperature of 100 °C and a pressure of 132 bar, 20 mL was used for a 10 min extraction. The resultant extract was transferred to a 500 mL glass bottle with a polytetrafluoroethylene lid for storage, and further extractions were conducted using the SAFE and HVE methods.

#### 2.5.2. Extraction of Key Volatile Flavor Compounds from Fried Tilapia Mince Using HVE

The initial extract obtained from ASE was added to the separatory funnel of the HVE device. The temperature of the super thermostat system was set to 40 °C to achieve constant temperature conditions in the reaction chamber and transfer tubes. The fore pump was activated, and once the system vacuum reached 400 Pa, the molecular sieve was turned on. When the system vacuum reached a rough vacuum of 10^−1^ Pa, the oil diffusion pump was initiated. After the system vacuum reached 10^−2^ Pa, liquid nitrogen was loaded into the Dewar flask, and the collection bottle was submerged in liquid nitrogen. Once the vacuum stabilized at 10^−3^ Pa, the high vacuum valve beneath the separatory funnel was slowly opened, allowing the liquid to flow gradually into the reaction chamber. After the liquid had completely transferred from the funnel to the reaction chamber, the high vacuum valve was closed, and the setup was maintained for 15 min to allow for full evaporation of the liquid in the reaction chamber. Finally, the vacuum from the oil diffusion pump was discontinued, allowing the pump temperature to return to room temperature before shutting down the fore pump and the molecular sieve. The liquid collected in the collection chamber was transferred to a 500 mL round-bottomed flask. Using a spiky distillation column with a length of 1000 mm and an internal diameter of 100 mm, the liquid was concentrated to 1 mL under a heating temperature of 45 °C and a circulating water temperature of 4 °C. The concentrated liquid was then transferred to a 1.5 mL headspace vial, and finally analyzed using gas chromatography.

#### 2.5.3. Extraction of Key Volatile Flavor Compounds from Fried Tilapia Mince Using SAFE

The method was adapted from the one described by Rui Wang [13], with minor modifications. The initial extract obtained from ASE was added to the separatory funnel of the SAFE device. The extraction was conducted under constant temperature conditions of 40 °C and a vacuum of 10^−3^ Pa. After the extraction was completed, the liquid collected in the collection chamber was transferred into a 500 mL round-bottomed flask. Using a spiky distillation column with a length of 1000 mm and an internal diameter of 100 mm, the liquid was concentrated to 1 mL under a heating temperature of 45 °C and a circulating water temperature of 4 °C. The concentrated liquid was then transferred to a 1.5 mL headspace vial and finally analyzed using gas chromatography.

#### 2.5.4. Extraction of Key Volatile Flavor Compounds from Fried Tilapia Mince Using SDE

Following a modified method described by Vilma Kraujalytė [14], 40 g of fried tilapia was weighed and added to a 500 mL round-bottomed flask, along with 200 g of saturated salt water, and the flask was connected to an SDE device. Subsequently, 50 mL of dichloromethane was added to a 100 mL round-bottomed flask, which was connected to the other end of the SDE setup. The dichloromethane was heated in a water bath to 58 °C until the liquid level in the SDE’s middle U-tube surpassed the bottom of the tube. A heater was then used to bring the dichloromethane with saturated salt water in the 500 mL flask to a gentle boil, with the condenser water temperature in the SDE set at 4 °C. After extracting for 4 h, the contents from the U-tube and the SDE were thoroughly collected in a 100 mL flask. To this, 5 g of anhydrous sodium sulfate was added and mixed well to fully absorb the moisture. The mixture was then transferred to a 500 mL round-bottomed flask. Under heating conditions of 45 °C and a circulating water temperature of 4 °C, the liquid was concentrated to 1 mL using a 1000 mm long, 100 mm internal diameter spiky-type distillation column. The concentrated liquid was then transferred to a 1.5 mL headspace vial and finally analyzed using gas chromatography.

### 2.6. Gas Chromatography (GC) Analysis of Volatile Compounds

Following a modified method described by Xie [15], gas chromatography was conducted using an Agilent 8890 with a flame ionization detector. The chromatographic column used was an HP-INNOWAX (60 m × 0.25 mm × 0.25 µm). The inlet temperature was set at 250 °C, and the injection mode was split, with an injection volume of 1 µL. High-purity nitrogen served as the carrier gas at a flow rate of 1 mL/min. The temperature program started at 40 °C, which was held for 3 min, then ramped at a rate of 5 °C/min to 75 °C and held for 3 min, followed by a ramp at 5 °C/min to 95 °C which was held for 3 min. It then increased at 2 °C/min to 115 °C and was held for 3 min, ramped at 3 °C/min to 165 °C and held for 3 min, and finally increased at 10 °C/min to 240 °C, where it was held for 35 min. The detector temperature was maintained at 280 °C, with an air flow of 450 mL/min, hydrogen flow of 40 mL/min, and makeup gas flow of 25 mL/min. The sampling frequency was 10 Hz. The external standard method was employed for the quantification of compounds. A calibration curve was established using the concentrations and peak areas of the reference standards, and the detected compounds were quantified based on this calibration curve.

### 2.7. Triangle Test Method to Assess the Effectiveness of Three Extraction Methods on the Aromatic Substances from Fried Tilapia Mince

Adapting the method slightly from Grigorakis [16], we conducted the triangle test to evaluate the sensory similarity of aroma substances extracted using three different methods, HVE, SAVE, and SDE, to the original tilapia. From each method, 100 µL of the extract was applied to qualitative filter paper, allowed to air dry for 20 s in a fume hood, and then sealed in plastic bags with each sample randomly coded. A reference sample of 10 g of fried tilapia was placed into a 100 mL polytetrafluoroethylene bottle.

Following the counterbalancing principle, thirteen trained panelists were provided with the reference sample and filter papers prepared from the three coded extracts during each session. Panelists were required to cleanse their mouths before each evaluation, rinse with water afterward, and rest for 30 s. After each evaluation, the panelists compared the aroma of the three samples to determine which was closest to the original tilapia, recording their evaluations. Even if uncertain, they were required to make a choice and record the similarity to the standard reference. Upon completion of all evaluations, significance analysis was performed using binomial distribution critical values to identify the extraction method that most closely replicated the sensory characteristics of the original tilapia.

The study was reviewed and approved by the Guangxi University IRB and informed consent was obtained from each subject prior to their participation in the study.

### 2.8. Data Analysis

All statistical analyses were performed using SPSS software version 23.0. Data are presented as mean ± SD (n = 3). Heatmaps were created using the R language package. Other images were processed using Origin software.

## 3. Results and Discussion

### 3.1. Precision and Reproducibility Results for the HVE Extraction Device

The data in Table 1 cover three nitrogen-containing compounds, two sulfur-containing compounds, five aldehydes, one ketone, and one lipid, totaling twelve compounds, with regression coefficients of standard curve equations all exceeding 0.999, suitable for quantitative analysis.

Nitrogen-containing compounds are key components in the formation of food flavors, playing a decisive role in regulating and shaping the overall flavor characteristics of food. Compounds such as 2,3,5-trimethylpyrazine, 2,3-dimethyl-5-ethylpyrazine, and 2,3-dimethylpyrazine contribute to complex and rich aromas such as earthy, nutty, and roasted in foods, significantly influencing consumer food choices and taste preferences [17]. During food processing, especially during high-temperature cooking, these compounds participate in flavor formation mechanisms such as the Maillard reaction due to their unique chemical properties, thereby generating distinctive flavor compounds. In this study, the quantitative analysis of 2,3,5-trimethylpyrazine, 2,3-dimethyl-5-ethylpyrazine, and 2,3-dimethylpyrazine demonstrated the accuracy and effectiveness of integrating high-vacuum extraction techniques with GC in the field of food chemical analysis. The concentrations of 2,3,5-trimethylpyrazine, 2,3-dimethyl-5-ethylpyrazine, and 2,3-dimethylpyrazine were found to be 24.15 ± 2.74 mg/L, 21.39 ± 2.78 mg/L, and 21.56 ± 2.73mg/L, respectively, showcasing the high precision and stability of the methods employed, with relative standard deviations of 11.36%, 12.99%, and 12.67%, indicating excellent experimental repeatability. The recovery rates for 2,3,5-trimethylpyrazine, 2,3-dimethyl-5-ethylpyrazine, and 2,3-dimethylpyrazine were 96.59%, 85.54%, and 86.24%, respectively. These high recovery rates not only illustrate the efficiency of the analytical process but also highlight the significant advantages of the method in ensuring minimal loss of nitrogen-containing compounds during extraction and analysis.

Sulfur-containing compounds are key constituents in many foods, significantly impacting the aroma characteristics of these products. These compounds, particularly 2-n-hexylthiophene and dimethyl trisulfide, due to their unique chemical properties, can significantly affect the overall flavor perception of foods even at very low concentrations [4]. They are widely present in vegetables, meats, and dairy products, contributing a range of flavors from pleasant freshness to undesirable spoilage. 2-n-hexylthiophene, especially found in coffee, meats, and certain fruits, provides distinctive nutty and roasted flavors, enhancing the food’s aroma. Through high vacuum extraction and gas chromatography analysis, this study accurately measured its concentration at 25.80 ± 2.86 mg/L, with a relative standard deviation of 11.08% and a recovery rate of 85.99%. Dimethyl trisulfide, predominantly found in thermally processed meats with a major flavor characteristic of roasted meat [18] was quantified at 22.30 ± 2.78mg/L, with a relative standard deviation of 12.45% and a recovery rate of 89.19%. These results once again confirm the efficiency and accuracy of the analytical methods, highlighting the critical role of high vacuum extraction combined with gas chromatography analysis in ensuring the precise quantification of these key sulfur-containing compounds.

In the food industry’s flavor analysis, aldehyde compounds play a crucial role; (E)-2-Octenal imparts a strong grassy odor [19], (E,E)-2,4-Decadienal a greasy aromatic scent [20], benzaldehyde an almond fragrance [21], trans-2-nonenal a cucumber and greasy odor [22], and hexanal grassy and fruity aromas [23]. Even at very low concentrations, these compounds significantly affect the overall flavor of food. This study combined HVE with gas chromatography analysis to investigate the presence of these compounds in food and their contributions to flavor, verifying the reproducibility and stability of the HVE equipment in scientific research. The results indicated that (E)-2-Octenal was present at 22.22 ± 1.98 mg/L with a relative standard deviation of 8.90% and a recovery rate of 88.86%; (E,E)-2,4-Decadienal was present at 22.38 ± 2.20 mg/L with a relative standard deviation of 9.85% and a recovery rate of 89.53%; benzaldehyde was present at 21.88 ± 2.51 mg/L with a relative standard deviation of 11.46% and a recovery rate of 87.53%; trans-2-nonenal was found at 24.71 ± 1.98 mg/L with a relative standard deviation of 8.01% and a recovery rate of 98.83%; and hexanal was present at 21.88 ± 2.95 mg/L with a relative standard deviation of 13.49% and a recovery rate of 87.54%. These findings underscore the application value of HVE technology in ensuring the reproducibility and stability of the extraction and analysis of key aldehyde compounds in food.

Ketone compounds such as hydroxyacetone can also significantly influence food flavors at trace levels, thus their role is crucial. Hydroxyacetone is often described as having a caramel or creamy flavor [24]. Experimental results indicated that the concentration of hydroxyacetone was determined to be 37.33 ± 4.84 mg/L, with a relative standard deviation of 12.97% and a recovery rate of 93.32%. These data robustly demonstrate the effectiveness of utilizing HVE technology to extract key ketone compounds and ensure the accuracy and reliability of the analysis.

Ester compounds such as D-pantolactone play a key role in the modulation and enhancement of food flavors due to their unique aromatic and taste properties. D-pantolactone, known for its mild creamy or cheesy aroma, is widely used in baked goods, dairy products, and condiments [25]. Experimental data indicated that the concentration of D-pantolactone was measured at 24.37 ± 3.41 mg/L, with a relative standard deviation of 14.01% and a recovery rate as high as 97.48%. These results effectively confirm the efficacy of utilizing HVE technology to extract key ester compounds and ensure the precision and reliability of the analysis.

### 3.2. Study on the Extraction Efficiency of Different Types of Volatile Compounds in Tilapia by Various Extraction Methods

In the study of extraction efficiency for different types of volatile compounds in tilapia using various extraction methods, GC-FID combined with external standard methods for gas chromatography was employed to quantitatively analyze fourteen types of volatile aromatic compounds in fried tilapia mince, including seven aldehyde compounds, four nitrogen-containing compounds, two sulfur-containing compounds, and one ester compound, comprehensively covering the key volatile compounds present in fried tilapia mince [1]. Table 2 presents the comparative data on the extraction efficiency of volatile compounds from fried tilapia mince using different extraction equipment. Through gas chromatography analysis, the regression coefficients of the standard curve equations obtained were all greater than 0.999, indicating that the equations have good applicability and are suitable for quantitative analysis.

**Figure 2:**
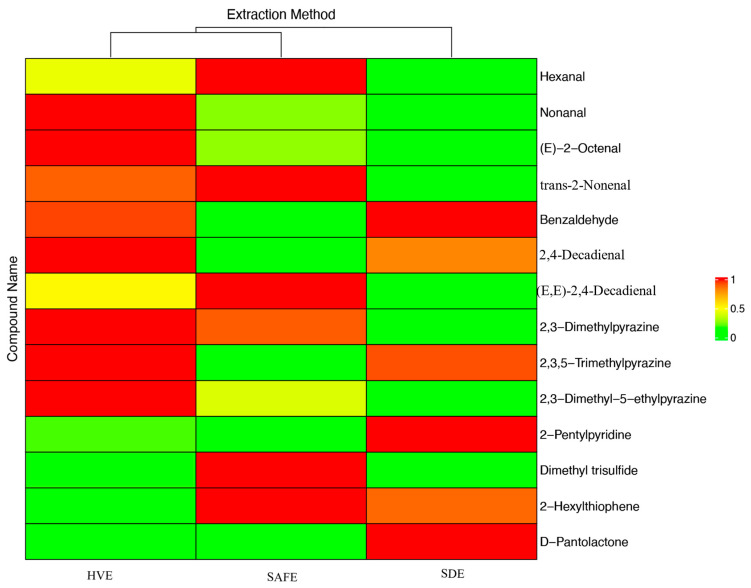
Heatmap of the extraction of volatile compounds from fried tilapia by three extraction devices.

Figure 2 displays a heatmap showcasing the extraction efficiencies of three different devices—high vacuum flavor extraction (HVE), solvent-assisted flavor evaporation (SAFE), and simultaneous distillation–extraction (SDE)—for volatile compounds in fried tilapia mince. In the cluster analysis represented in Heatmap 4, HVE and SAFE are grouped together, reflecting their shared utilization of high-vacuum techniques to extract volatile flavor compounds. In the heatmap, shades closer to red indicate higher concentrations of these compounds, whereas shades approaching green denote lower concentrations. The data reveal that, relative to the other methods, HVE demonstrates a pronounced efficacy in extracting aldehyde and pyrazine compounds. However, it appears to be less effective in extracting sulfur and lipid compounds when compared to SDE. This conclusion is similar to the findings of Majcher et al. in 2009 [7], who compared the applicability of SPME, SAFE, and SDE methods in extracting flavor compounds from extruded potato snacks, noting that SDE’s high temperature and prolonged extraction could produce more lipids and sulfur-containing compounds. However, the specific reasons still require further analysis based on each type of compound.

For hexanal and (E,E)-2,4-decadienal, significant differences in extraction results were observed among the three methods (*p* < 0.05), with SAFE performing relatively better, whereas SDE was less effective than the other two methods. For nonanal and (E)-2-octenal, HVE showed significant differences compared to the other two methods (*p* < 0.05), whereas the differences between SAFE and HVE were not significant (*p* > 0.05), indicating that HVE was more effective in extracting these two compounds. The extraction results for trans-2-nonenal showed no significant differences between HVE and SAFE (*p* > 0.05), whereas SDE yielded significantly less trans-2-nonenal than the other two methods (*p* < 0.05). For benzaldehyde, there were no significant differences between HVE and SDE (*p* > 0.05), but SAFE obtained significantly less benzaldehyde than the other two methods (*p* < 0.05). For 2,4-decadienal, all three methods showed significant differences (*p* < 0.05), with HVE performing the best and SDE the worst. Overall, for the extraction of aldehyde compounds, SDE was clearly less effective than the two high vacuum extraction methods, possibly due to the ease of extraction of aldehyde volatile compounds under high vacuum conditions, as mentioned by Jia Huang et al. in 2018 [26]. This may be because the high-temperature, long-duration operating conditions can produce more compounds. SDE also shows good extraction results for aldehyde compounds, particularly notable in the extraction of benzaldehyde, but the extraction of flavor substances in long-term high-temperature environments by SDE may generate “pseudo flavor substances”, a conclusion similar to the findings of Amanpour et al. in 2015 [27] and Gu et al. in 2023 [28].

In the extraction of 2,3-dimethylpyrazine, there were no significant differences between the HVE and SAFE methods (*p* > 0.05), whereas the performance of SDE was significantly lower than the other two methods (*p* < 0.05). For the extraction of 2,3,5-trimethylpyrazine, 2,3-dimethyl-5-ethylpyrazine, and dimethyl trisulfide, no significant differences were observed among the three methods (*p* > 0.05). The results indicate that the three methods have similar extraction efficiencies for pyrazine compounds and sulfur-containing compounds, suggesting that all three methods are effective for the extraction of pyrazine compounds. However, for 2,3-dimethylpyrazine, a pyrazine compound with a lower boiling point and thermal sensitivity, the two high vacuum extraction methods demonstrated greater advantages. This is consistent with the findings of M. Majcher et al. in 1999 [29].

For 2-Pentylpyridine, there were significant differences among the three methods (*p* < 0.05), with SDE showing the best extraction effect, HVE ranking second, and SAFE performing the worst, which may be because 2-Pentylpyridine is an important component in heat-processed foods and the prolonged high temperature of the SDE extraction could result in the release of more 2-Pentylpyridine [28]. For 2-hexylthiophene, the extraction performance of SAFE was significantly lower than that of SDE and HVE (*p* < 0.05), with no significant differences between the latter two, indicating that SDE and the two high vacuum extraction methods can effectively extract sulfur compounds [30], yet SAFE performed less effectively for 2-hexylthiophene. For the ester compound D-pantolactone, there were no significant differences between HVE and SAFE (*p* > 0.05), but the performance of SDE was significantly higher than the other two methods (*p* < 0.05). This conclusion indicates that SDE was more effective for extracting ester compounds from tilapia. However, due to the higher boiling point of lipid-like compounds, SDE extracted the most D-pantolactone, which may be related to the generation of more lipid compounds during the high-temperature extraction process [31].

Due to the presence of numerous fine insoluble substances in extracts from fried foods using solvent extraction, neither SAFE (solvent-assisted flavor evaporation) nor HVE (high vacuum evaporation) can be directly employed for further extraction. These small solid particles can clog the high vacuum valves used for adding liquids, preventing the sample from being introduced into the machine. Moreover, if the liquid obtained directly from ASE (accelerated solvent extraction) is used, the significant amount of fats dissolved in the solvent form azeotropes with the solvent and volatile compounds. This leads to increased concentration temperatures and prevents concentration to the desired volume, resulting in substantial losses of volatile compounds during the concentration process. Therefore, after several trials, we opted for ASE to obtain an initial extract devoid of solid material but containing many high-boiling compounds (such as fats). Subsequently, HVE or SAFE is used to extract volatile compounds, effectively separating out high-boiling components (such as fats).

### 3.3. Comparison of Odor Profiles of Volatile Compounds in Fried Tilapia Mince by Three Different Extraction Methods

**Figure 3:**
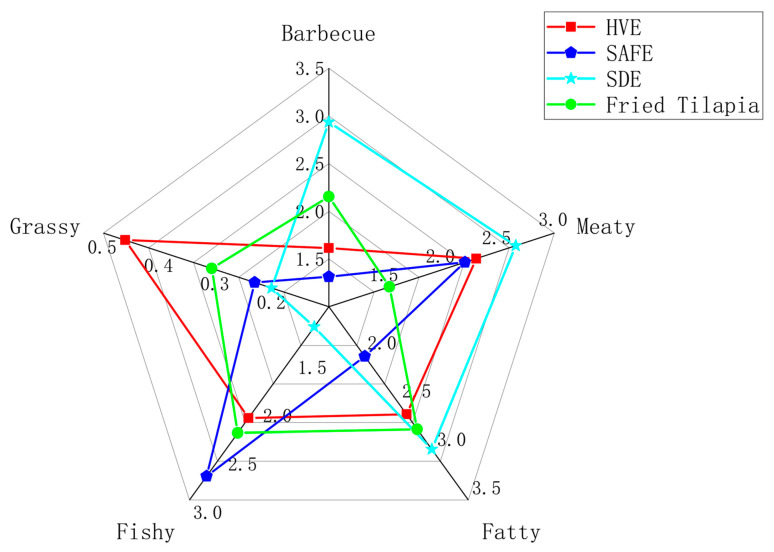
Odor profile of volatile compounds in fried tilapia using three different extraction methods. HVE: high vacuum flavor extraction, SAFE: solvent-assisted flavor evaporation, SDE: simultaneous distillation–extraction.

Figure 3 displays the comparison of odor profiles of volatile compounds in fried tilapia mince extracted by three different methods. The main odor profiles of fried tilapia mince include barbecue, grassy, fishy, fatty, and meaty aromas [1]. The comparison results show that in the barbecue profile, the odor similarity percentages to the original sample for the high vacuum extraction method, solvent-assisted evaporation method, and simultaneous distillation–extraction method were 75.00%, 61.07%, and 63.84%, respectively; in the grassy profile, they were 50.00%, 56.50%, and 27.23%; in the fishy aroma, they were 93.33%, 80.67%, and 52.39%; in the fatty aroma, they were 94.44%, 72.78%, and 92.49%; and in the meaty aroma, they were 50.00%, 56.50%, and 27.23%. Combining the similarity percentages for each aroma and assuming equal importance of each in the overall odor profile, the average overall odor similarities for the high vacuum extraction method, solvent-assisted evaporation method, and simultaneous distillation–extraction method of the original sample were 72.55%, 69.25%, and 60.29%, respectively. Thus, the extract obtained by the high vacuum extraction method most closely resembled the original odor profile of fried tilapia mince. Simultaneous distillation–extraction, due to prolonged high-temperature operation, may lead to the production of an excess of aromatic substances, enhancing meat, fat, and barbecue flavors, and thus resulting in a larger deviation from the original sample’s odor profile. Sarhir et al. in 2019 found that SAFE extracted more representative flavor substances during their study of Ayran aroma-active compounds [32]. Lingyun Yao et al. in 2021, using SDE, SAFE, and SPME methods to extract volatile compounds from dried Xinjiang figs, reported that the number of aroma-active compounds identified by SAFE, SPME, and SDE were 49, 36, and 47, respectively. Although SDE detected many compounds, these compounds were only detectable by SDE. In the SDE process, the sample is heated, which can accelerate the release of volatile compounds in figs, but the increased temperature might also lead to the Maillard or Strecker reactions creating a false impression, thereby promoting the accumulation of non-genuine aroma compounds or increasing the concentration of genuine aroma compounds, thus altering the compound’s odor profile [33]. These findings are consistent with the significant changes in the odor profile observed with SDE in this experiment. The experimental results indicate that HVE provides the best extraction performance for volatile compounds in fried tilapia mince.

## 4. Conclusions

The findings of this study indicate that HVE demonstrates excellent precision and reproducibility, effectively addressing the issues of artifact generation and high maintenance costs associated with traditional extraction methods. Compared to the SAFE and SDE extraction methods, HVE not only aids in the extraction and recovery of volatile compounds from fried tilapia mince but also yields volatile extracts that most closely resemble the odor profile of fried tilapia mince. Lastly, although HVE as a novel extraction technique may still require further refinement, it undoubtedly provides a new avenue for research into food flavors and food quality control.

## Figures and Tables

**Table 1:** HVE extraction equipment precision reproducibility experimental data table.

SN	CC	Compound Name	Standard Curve Equation	RC	Content (mg/L)	RSD (%)	RR (%)
1	Nitrogen Compounds	2,3,5-Trimethylpyrazine	y = 28.23 + 12.57x	0.9992	24.15 ± 2.74	11.36	96.59
2		2,3-Dimethyl-5-ethylpyrazine	y = 36.64 + 13.17x	0.9991	21.39 ± 2.78	12.99	85.54
3		2,3-Dimethylpyrazine	y = 30.37 + 12.76x	0.9993	21.56 ± 2.73	12.67	86.24
4	Sulfur Compounds	2-Hexylthiophene	y = 32.90 + 16.20x	0.9994	25.80 ± 2.86	11.08	85.99
5		Dimethyl trisulfide	y = 11.47 + 3.32x	0.9992	22.30 ± 2.78	12.45	89.19
6	Aldehydes	(E)-2-Octenal	y = 30.82 + 12.37x	0.9991	22.22 ± 1.98	8.90	88.86
7		(E,E)-2,4-Decadienal	y = 22.97 + 9.35x	0.9997	22.38 ± 2.20	9.85	89.53
8		Benzaldehyde	y = 10.05 + 6.48x	0.9995	21.88 ± 2.51	11.46	87.53
9		trans-2-Nonenal	y = 20.36 + 11.39x	0.9994	24.71 ± 1.98	8.01	98.83
10		Hexanal	y = 27.85 + 9.58x	0.9990	21.88 ± 2.95	13.49	87.54
11	Ketones	Hydroxyacetone	y = 5.99x + 0.37	0.9997	37.33 ± 4.84	12.97	93.32
12	Esters	D-Pantolactone	y = −3.59 + 2.57x	0.9993	24.37 ± 3.41	14.01	97.48

SN: serial number, CC: compound category, RC: regression coefficient, RSD: relative standard deviation, RR: recovery rate.

**Table 2:** Comparative data table on the extraction of volatile compounds in fried tilapia using three types of extraction equipment.

SN	CC	Compound Name	Standard Curve Equation	RC	HVE (mg/kg)	SAFE (mg/kg)	SDE (mg/kg)
1	Aldehydes	Hexanal	y = 68.57x − 1.57	0.9998	60.48 ± 7.41 ^a^	99.30 ± 11.82 ^b^	27.72 ± 1.43 ^c^
2		Nonanal	y = 6.68x + 0.28	0.9990	258.16 ± 36.47 ^a^	225.63 ± 5.29 ^b^	213.99 ± 7.87 ^b^
3		(E)-2-Octenal	y = 8.82x + 1.02	0.9994	47.065 ± 6.687 ^a^	27.19 ± 1.91 ^b^	19.51 ± 3.19 ^b^
4		trans-2-Nonenal	y = 37.46x + 1.81	0.9996	55.09 ± 5.29 ^a^	58.74 ± 8.36 ^a^	22.81 ± 1.76 ^b^
5		Benzaldehyde	y = 43.16x + 0.38	0.9998	131.61 ± 14.67 ^a^	29.77 ± 2.02 ^b^	137.70 ± 12.20 ^a^
6		2,4-Decadienal	y = 26.18x − 0.23	0.9995	60.80 ± 7.49 ^a^	9.09 ± 0.79 ^b^	51.511 ± 3.272 ^c^
7		(E,E)-2,4-Decadienal	y = 18.40x + 2.92	0.9993	341.05 ± 35.59 ^a^	477.51 ± 68.90 ^b^	195.08 ± 0.65 ^c^
8	Nitrogen Compounds	2,3-Dimethylpyrazine	y = 30.27x + 1.70	0.9997	35.76 ± 3.82 ^a^	33.57 ± 1.82 ^a^	12.25 ± 1.05 ^b^
9		2,3,5-Trimethylpyrazine	y = 33.32x + 2.01	0.9998	34.81 ± 4.55 ^a^	27.65 ± 3.73 ^a^	34.30 ± 5.12 ^a^
10		2,3-Dimethyl-5-ethylpyrazine	y = 32.41x + 2.56	0.9998	26.61 ± 1.83 ^a^	25.07 ± 1.65 ^a^	23.98 ± 3.06 ^a^
11		2-Pentylpyridine	y = 39.95x + 1.66	0.9997	53.82 ± 8.21 ^a^	2.35 ± 0.38 ^b^	265.29 ± 32.61 ^c^
12	Sulfur Compounds	Dimethyl Trisulfide	y = 8.82x + 1.02	0.9994	60.65 ± 10.00 ^a^	77.35 ± 9.33 ^a^	57.18 ± 3.16 ^a^
13		2-Hexylthiophene	y = 13.09x − 0.23	0.9995	5.55 ± 0.37 ^a^	23.29 ± 3.15 ^b^	21.22 ± 0.10 ^b^
14	Esters	D-Pantolactone	y = 21.20x − 1.09	0.9999	126.09 ± 9.64 ^a^	128.30 ± 3.07 ^a^	197.48 ± 24.71 ^b^

SN: serial number, CC: compound category, RC: regression coefficient, HVE: high vacuum flavor extraction, SAFE: solvent-assisted flavor evaporation, SDE: simultaneous distillation–extraction, ^a–c^ Different letters in the same row indicate significant differences (*p* < 0.05).

## Data Availability

The original contributions presented in the study are included in the article, further inquiries can be directed to the corresponding author.
